# Identification of upregulated NF-κB inhibitor alpha and IRAK3 targeting lncRNA following intracranial aneurysm rupture-induced subarachnoid hemorrhage

**DOI:** 10.1186/s12883-021-02156-1

**Published:** 2021-05-14

**Authors:** Wei Leng, Dan Fan, Zhong Ren, Qiaoying Li

**Affiliations:** Department of Neurology, Affiliated Hospital of Changchun University of Traditional Chinese Medicine, Changchun, 130021 Jilin China

## Abstract

**Background:**

This study was performed to identify genes and lncRNAs involved in the pathogenesis of subarachnoid hemorrhage (SAH) from ruptured intracranial aneurysm (RIA).

**Methods:**

Microarray GSE36791 was downloaded from Gene Expression Omnibus (GEO) database followed by the identification of significantly different expressed RNAs (DERs, including lncRNA and mRNA) between patients with SAH and healthy individuals. Then, the functional analyses of DEmRNAs were conducted and weighted gene co-expression network analysis (WGCNA) was also performed to extract the modules associated with SAH. Following, the lncRNA-mRNA co-expression network was constructed and the gene set enrichment analysis (GSEA) was performed to screen key RNA biomarkers involved in the pathogenesis of SAH from RIA. We also verified the results in a bigger dataset GSE7337.

**Results:**

Totally, 561 DERs, including 25 DElncRNAs and 536 DEmRNAs, were identified. Functional analysis revealed that the DEmRNAs were mainly associated with immune response-associated GO-BP terms and KEGG pathways. Moreover, there were 6 modules significantly positive-correlated with SAH. The lncRNA-mRNA co-expression network contained 2 lncRNAs (LINC00265 and LINC00937) and 169 mRNAs. The GSEA analysis showed that these two lncRNAs were associated with three pathways (cytokine-cytokine receptor interaction, neurotrophin signaling pathway, and apoptosis). Additionally, *IRAK3* and *NFKBIA* involved in the neurotrophin signaling pathway and apoptosis while *IL1R2*, *IL18RAP* and *IL18R1* was associated with cytokine-cytokine receptor interaction pathway. The expression levels of these genes have the same trend in GSE36791 and GSE7337.

**Conclusion:**

LINC00265 and LINC00937 may be implicated with the pathogenesis of SAH from RIA. They were involved in three important regulatory pathways. 5 mRNAs played important roles in the three pathways.

**Supplementary Information:**

The online version contains supplementary material available at 10.1186/s12883-021-02156-1.

## Background

Subarachnoid hemorrhage (SAH) is an acute, devastating hemorrhagic stroke accounting for 5% of cerebrovascular strokes [1, 2]. SAH from ruptured intracranial aneurysm (RIA) is a non-traumatic type SAH with destructive central nervous system, and has a high in-hospital mortality (45%) [3–5], disability rate (30%), and high morbidity of long-term cognitive impairment (50%) among survivors [6, 7]. Delayed cerebral ischemia and vasospasm following SAH are the primary causes of SAH-induced mortality in intensive care unit [8, 9]. Although drug therapy, such as intrathecal nicardipine and statins, has showed some effect on reducing SAH-induced mortality, the overall clinical outcomes has been unsatisfactory due to drug-related myotoxicity and side effect on liver and kidney [10–12]. Therefore, it is imperative to gain comprehensive understanding of the pathogenesis of SAH from RIA, which will greatly promote to develop effective therapeutic strategies against this disease.

The transcriptome profiling analysis provides new opportunities for identifying promising therapeutic targets and exploring the molecular mechanisms of various diseases. Notably, increasing researchers have focused on elaborating the underlying pathological mechanisms of SAH by the transcriptomic analysis. For example, Pera et al suggested that SAH from RIA significantly influences the gene expression profiles of peripheral blood cells and 16 transcriptional biomarkers could differentiate IRA patients and healthy individuals by a microarray analysis [13]. Lai et al performed a miRNA microarray analysis and demonstrated that miR-4320 may be a potentially valuable signatures for the detection of SAH [14]. Additionally, Liang et al reported that the expression of a long non-coding RNA (lncRNA) MEG3 was positively correlated with the severity of SAH [15]. They also showed that lncRNA MEG3 inhibited the neuron activity through the PI3K/Akt signaling pathway. Although several RNA biomarkers have been showed to be associated with SAH, the potential mechanism of SAH from RIA have not been fully understood.

We performed a co-expression analysis to identify potential mRNA and lncRNA signatures involved in the pathogenesis of SAH from RIA. The differential expression analysis was carried out and then subjected to a subsequent functional analyses RNAs makers involved in the pathogenesis of SAH from RIA. This study might provide a deeper insight into the molecular mechanisms of SAH from RIA.

## Methods

### Affrymetrix microarray dataset and standard processing

Microarray dataset GSE36791 (including lncRNA and mRNA) was downloaded from the National Center for Biotechnology Information-Gene Expression Omnibus (NCBI-GEO) database (https://www.ncbi.nlm.nih.gov/geo/) [13], it was generated by GPL10558 Illumina HumanHT-12 V4.0 expression beadchip platform (https://www.ncbi.nlm.nih.gov/geo/query/acc.cgi?acc=GPL10558), and includes 61 peripheral blood samples (18 healthy controls and 43 patients with SAH from RIA).

Afterwards, Limma package (version 3.34.0; https://bioconductor.org/packages/release/bioc/html/limma.html) [16] in R 3.4.1 was employed to preprocess raw data. First, we log2 the expression profile data to transform the gene expression data from a skewed distribution to an approximate normal distribution, and then normalize the data with the median standardization method.

### Identification of differentially expressed RNAs (DERs) and functional analyses

Firstly, The lncRNAs and mRNAs in GSE36791 datasetes were re-annotated using the HUGO Gene Nomenclature Committee (HGNC) database, consisting of annotated 4055 lncRNAs and 19,198 protein coding genes [17]. Then, the significantly DERs (, including lncRNA and mRNA) between SAH group and normal controls were identified using Limma package (version 3.34.0, https://bioconductor.org/packages/release/bioc/html/limma.html) in R3.4.1, with the threshold of false discovery rate (FDR) < 0.05 and |logFC| > 0.5. Afterwards, the bidirectional clustering analysis of DEGs was also conducted using R pheatmap package (version 1.0.8; https://cran.r-project.org/package=pheatmap) [18].

Finally, the analysis of Gene Ontology biological processes (GO-BP) and Kyoto Encyclopedia of Genes and Genomes (KEGG) pathways was carried out for those significant DERs using the web-accessible the Database for Annotation, Visualization and Integrated Discovery (DAVID, version 6.8; https://david.ncifcrf.gov/) software according to the cutoff criterion of FDR < 0.05 [19, 20].

### Weighted gene co-expression network analysis (WGCNA)

WGCNA is a tool to construct networks and identify gene clusters or modules of gene with co-expression profiling. Here, we carried out a WGCNA analysis to identify the modules associated with SAH using WGCNA integrated algorithm (version 1.61; https://cran.r-project.org/web/packages/WGCNA/index.html) in R 3.4.1 [21]. We set that each RNA module contains at least 30 RNA elements (cutHeight = 0.995). Then by calculating the Pearson correlation coefficient between the module eigengene (ME) of each module and each sample group, we selected modules that are significantly related to the disease. The selection criteria are as follows: 1) *P* value was less than 0.05; 2) the correlation coefficient was higher than that in the control (grey) module.

### Construction of lncRNA-mRNA regulatory network

The R cor function (http://77.66.12.57/R-help/cor.test.html) was employed to compute the Pearson correlation coefficient (PCC) between DElncRNAs and DEmRNAs in significant target modules. Thereby, the DElncRNAs-DEmRNAs co-expression network was established and visualized by Cytoscape software (version 3.6.1; http://www.cytoscape.org/) [22].

### Gene set enrichment analysis (GSEA) for RNAs in regulatory network

To further screen hub genes involved pathogenesis of SAH from RIA, the KEGG enrichment pathway analysis of genes which exhibited co-expression relationships with DElncRNAs was performed by GSEA (http://software.broadinstitute.org/gsea/index.jsp), which is a popular tool for interpreting the effects of collective behavior of genes on observed phenotypes by evaluating the deviation of gene expression between disease groups and healthy control group [23]. The cutoff of *P* < 0.05 was chosen as a statistically significant threshold.

### Expression level analysis for hub gens

In the GEO database, we searched a new data set (GSE7337) containing more samples to verify our results. There were 119 peripheral blood samples from SAH patients and 118 controls. The gene expression profiles were determined by Illumina HumanHT-12v4 BeadChips. We extracted the expression levels of hub genes in these two data sets and compared the expression in different samples.

## Results

### DERs identification and functional analyses

**Fig. 1:**
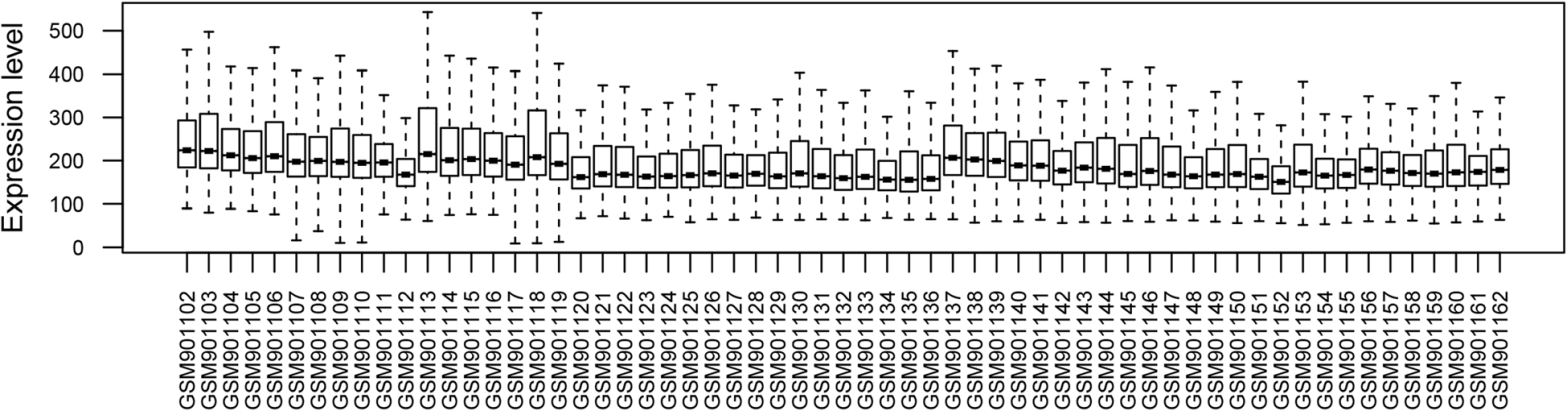
The boxplots of the data downloaded from GEO database. Gene expression level was analyzed using log2 transformation. Data before normalization

**Fig. 2:**
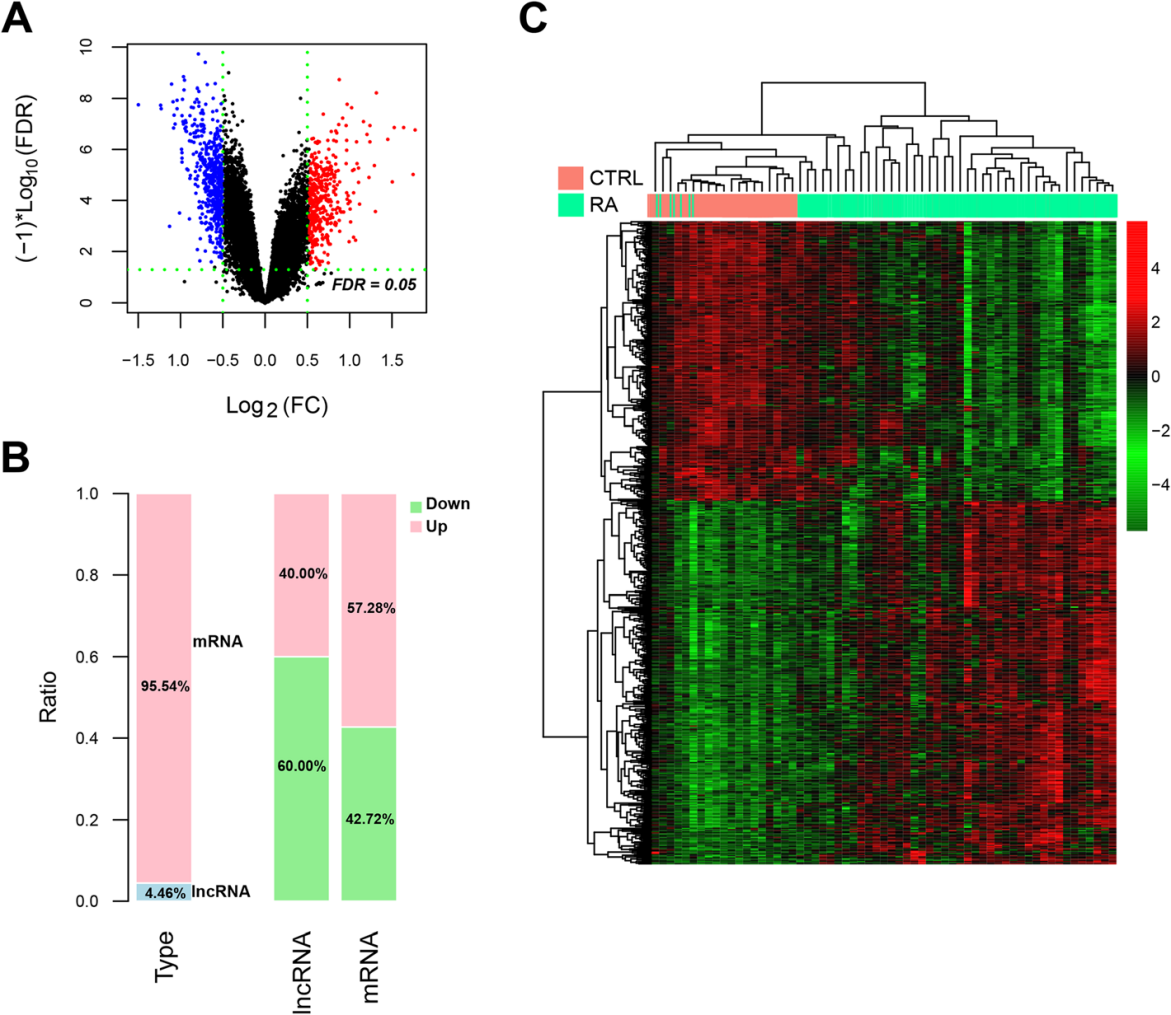
Statistics and expression of the significantly differentially expressed genes (DEGs) in SAH samples. **a** the Volcano plot of the SARs in SAH samples. Green lines indicate false discovery rate (FDR) =0.05 (horizontal line) and |log_2_[Fold change (FC) of FPKM] | = 0.5 (vertical lines), respectively. **b** the statistics of the DERs and percentage of lncRNAs and mRNAs, as well as down- and up-regulated DERs. **c** heatmap of the DERs in 43 SAH samples and 18 control (CTRL) samples

**Fig. 3:**
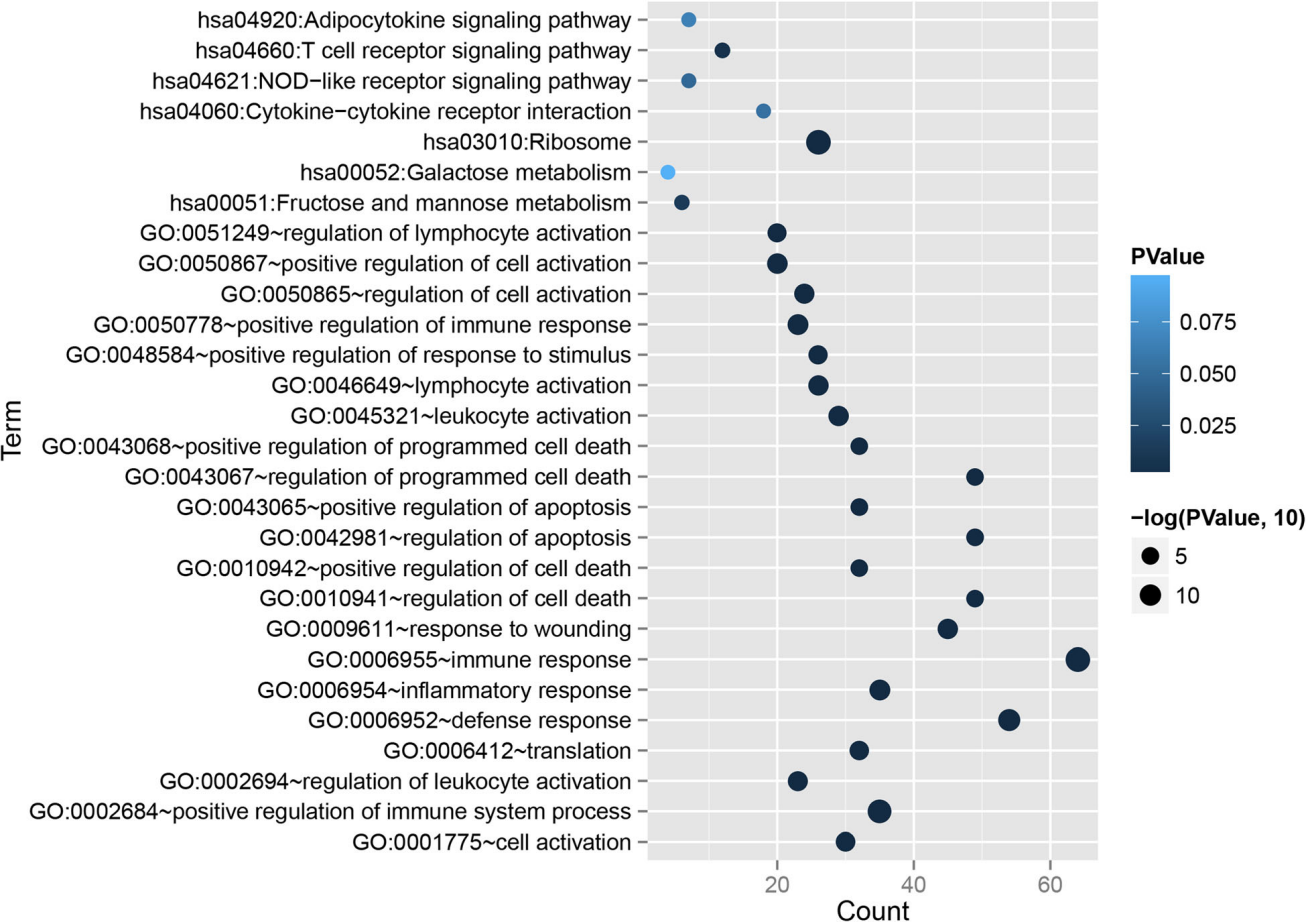
The ingenuity pathways and GO categories associated with significantly differentially expressed mRNAs (DEmRNAs) in SAH. 7 KEGG pathways and 21 GO biological processes were identified to be associated with 536 significantly DEmRNAs. Black to blue note *p* value close to 0 and 1.0 respectively. Bubbles indicate –log (*p* value). The larger the bubble, the higher –log (*p* value)

First, we need to standardize the downloaded expression profile data set (Fig. 1). Totally, 1053 lncRNAs and 18,320 protein coding RNAs were identified by annotating with HGNC database after data standardization. Moreover, 561 DERs were identified, including 25 DElncRNAs (10 up-regulated lncRNAs and 15 down-regulated lncRNAs) and 536 DEmRNAs (229 down-regulated mRNAs and 307 up-regulated mRNAs) according to the criteria of FDR < 0.05 and |logFC| ≥ 0.5 (Supplementary Table S1; Fig. 2a and b), with the criteria of FDR < 0.05 and |logFC| ≥ 0.5. Hierarchical clustering showed the distinct expression profiles of these DERs in the SAH from RIA samples and in controls (Fig. 2c). Additionally, functional enrichment analyses of DEmRNAs showed there were 21 GO-BP terms and 7 KEGG significant pathways. Briefly, these genes were mainly associated with GO-BP terms immune response, defense response, inflammatory response, leukocyte activation and regulation. The KEGG analysis indicated that these genes played essential roles in T cell receptor signaling pathway, NOD-like receptor signaling pathway, Cytokine-cytokine receptor interaction as well as Adipocytokine signaling pathway (Fig. 3).

### Construction and analysis of WGCNA modules associated with SAH traits

**Fig. 4:**
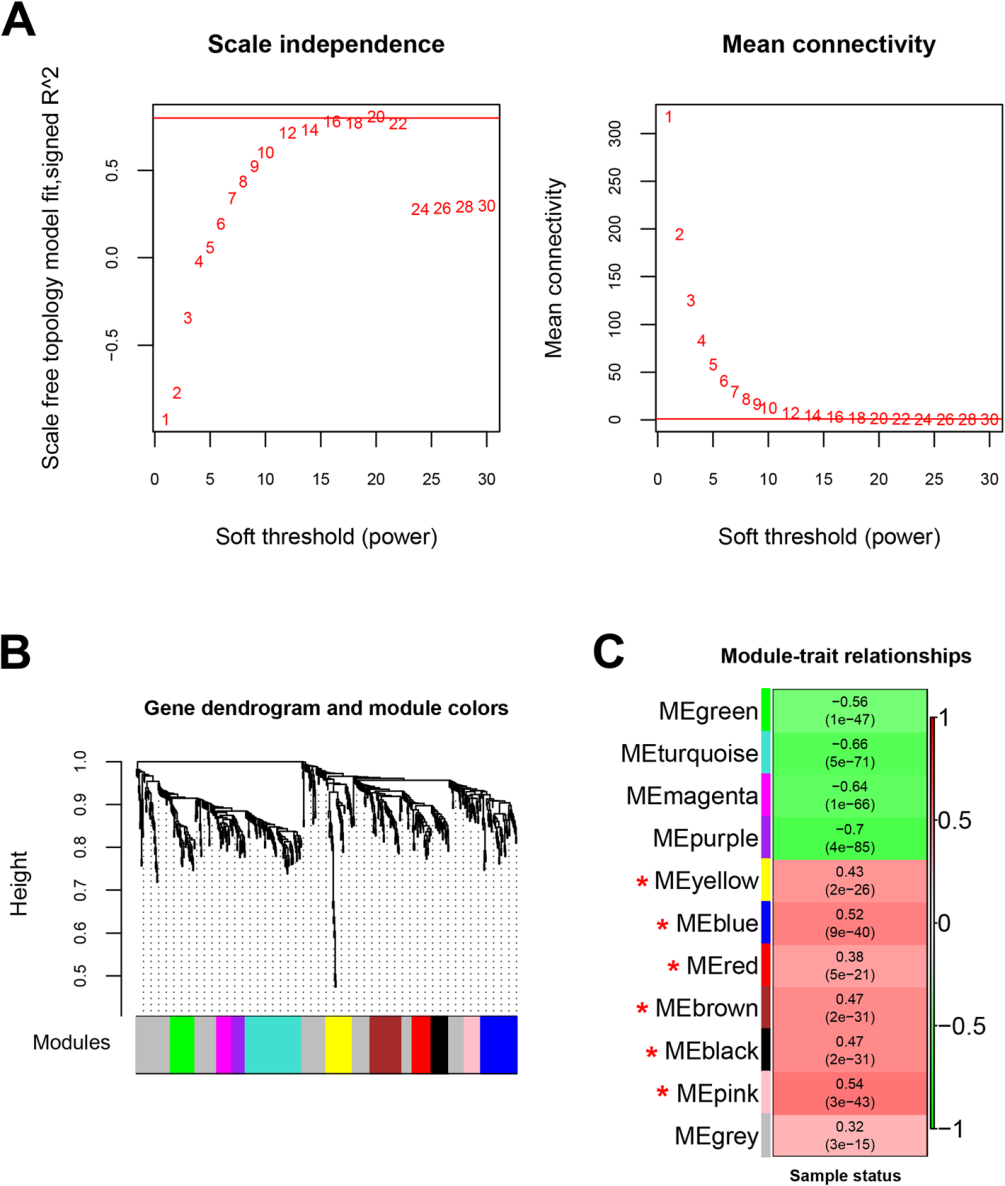
The construction of WGCNA modules in dataset GSE36791. **a** the correlation coefficient square (r^2^) of eigengenes (red line) under soft threshold power (red number). **b** the mean connectivity of eigengenes (red line) under soft threshold power (red number). **c** WGCNA modules in the gene dendrogram based on the criteria of number of genes > 100, cut Height = 0.995, soft threshold power 20 and *r*^2^ = 0.8. D, the heatmap showing the correlation of WGCNA modules with SAH clinical traits. Green and red color note negative and positive correlation, respectively. Red stars note 6 modules showed higher, closer correlation (correlation *p* value ≤0.05 and coefficient > 0.32) than gray module

All the 561 DERs were selected for the WGCNA module analysis. When we choose the parameters of an adjacency function, we only consider those parameter values that cause the network to only approximately satisfy the scale-free topology, such as R ^ 2 > 0.80. First, we need to consider average connectivity. It can determine whether the network can contain enough information such as module detection. Second, the slope of the regression line should be around − 1. In this research, the first parameter value was 0.8. the threshold of soft was power 20 (Fig. 4a). The mean connectivity reached 1 when soft-thresholding power was 20 (Fig. 4b), revealing WGCNA modules were constructed using the approximate scale-free topology. Then, the dissimilarity coefficients between gene nodes were calculated and the clustering tree was built. Accordingly, a total of nine WGCNA modules were constructed in the gene dendrogram (Fig. 4c), with the criteria of eigengenes number ≥ 100 genes, cut height = 0.995. Then we calculated the correlation between modules and status of each sample: disease or not (RIA or CTRL, Supplementary Table S2). The results revealed that six modules significantly positively correlated with disease, including yellow (*P*-value = 2e-26), blue (*P*-value = 9e-40), red (*P*-value = 5e-21), brown (*P*-value = 2e-31), black (*P*-value = 2e-31) and pink (*P*-value = 3e-43) modules. The list of the 217 DERs (2 lncRNAs and 215 mRNAs) included in the six WGCNA modules is shown in Supplementary Table S3.

### Construction of lncRNA and mRNA regulatory network

**Fig. 5:**
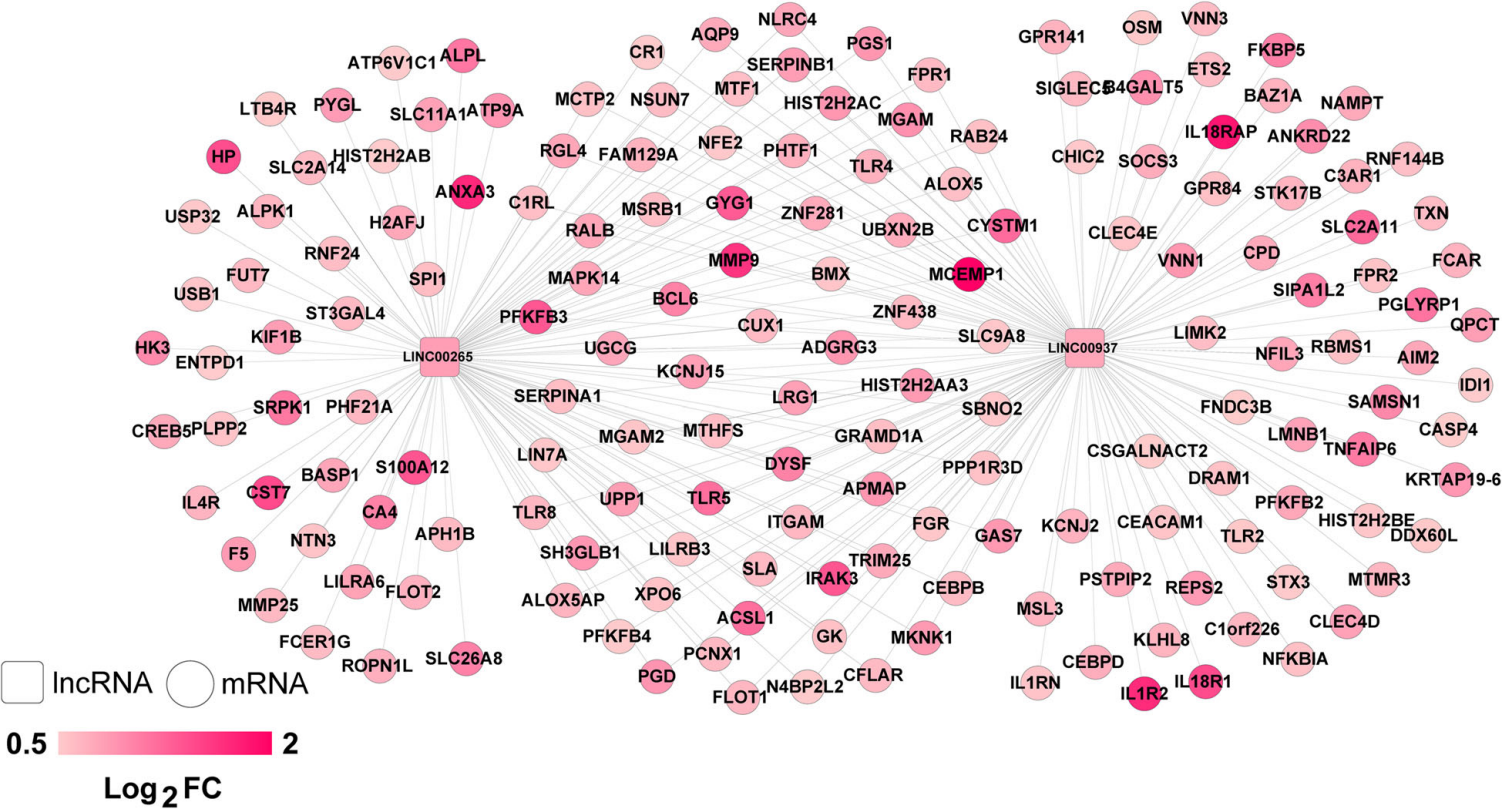
The lncRNA and mRNA regulatory network. Box and circle represent lncRNA and mRNA, respectively. Red color notes the upregulation in SAH relative to control

We calculated the PCC between DElncRNA and DEmRNA in the 6 WGCNA modules and retained the connection pairs with PCC higher than 0.6. A total of 240 pairs meet the requirements (Supplementary Table S4). They were composed of 171 nodes. Then the lncRNA and mRNA regulatory network was constructed (Fig. 5). All the notes including LINC00265 and LINC00937 were upregulated in SAH patients from RIA. We found that there were strong relationships between LINC00265 and two mRNAs (nuclear factor kappa B inhibitor alpha, *NFKBIA* and interleukin 1 receptor associated kinase 3, *IRAK3*). In addion, LINC00937 closely interacts with *NFKBIA*.

### GSEA for the lncRNAs in regulatory network

**Fig. 6:**
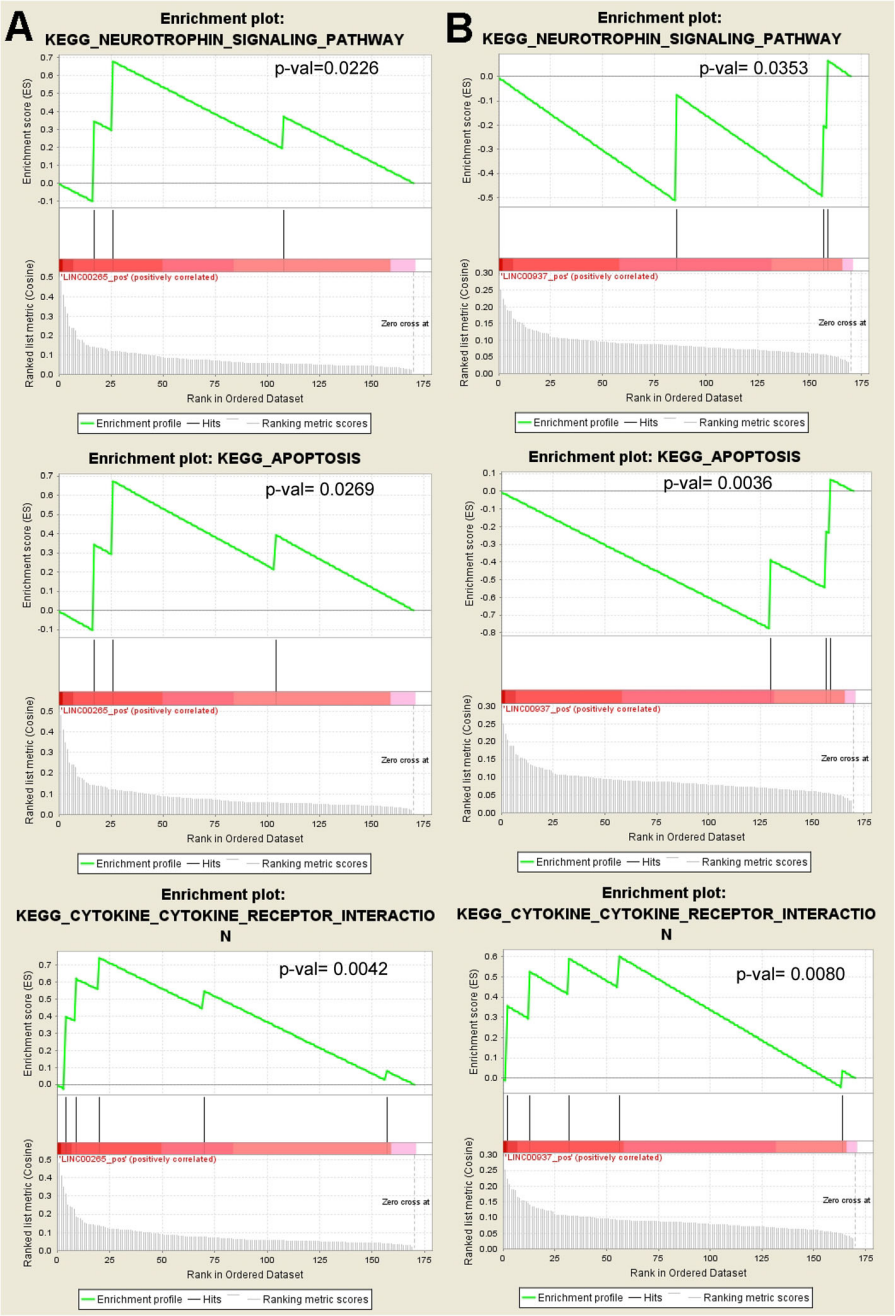
Overlapping KEGG pathways associated with both LINC00265 and LINC00937. KEGG pathways were identified using GSEA software and the positive correlations of lncRNA expression with pathway activation were confirmed

At last, we performed the GSEA to identify the KEGG pathways associated with the 169 mRNAs genes connected LINC00265 and LINC00937 in regulatory network as mentioned above. The results showed that a total of four KEGG pathways were associated with LINC00265, including cytokine-cytokine receptor interaction (*P*-value = 0.0042), neurotrophin signaling pathway (*P*-value = 0.0226), apoptosis (*P*-value = 0.0269) and neuroactive ligand receptor interaction (*P*-value = 0.0309). Five KEGG pathways were associated to LINC00937, including apoptosis (*P*-value = 0.0036), Toll like receptor signaling pathway (*P*-value = 0.0178), neurotrophin signaling pathway (*P*-value = 0.0353), cytokine-cytokine receptor interaction (*P*-value = 0.0080) and MAPK signaling pathway (*P*-value = 0.0489) (Table 1). Moreover, three overlapping KEGG pathways (‘Cytokine-Cytokine receptor interaction’, ‘Neurotrophin signaling pathway’, and ‘Apoptosis’) were positively correlated with LINC00265 and LINC00937 (Fig. 6). Two genes (*NFKBIA* and *IRAK3*) were overlapped in apoptosis and neurotrophin signaling pathway, respectively. Meanwhile, three overlapped genes, including interleukin 1 receptor type 2 (*IL1R2*), interleukin 18 receptor accessory protein (*IL18RAP*) and interleukin 18 receptor 1 (*IL18R1*) were involved in cytokine-cytokine receptor interaction.

**Table 1:** KEGG pathways associated with LINC00265 and LINC00937 and the target genes

Name	SIZE	ES	NES	NOM ***p***-val	Gene
*LINC00265*					
Cytokine-cytokine receptor interaction	3	0.7413	1.5656	0.0042	IL4R
Neurotrophin signaling pathway	2	0.6761	1.2246	0.0226	IRAK3,MAPK14
Apoptosis	2	0.6731	1.2001	0.0269	IRAK3, CFLAR
Neuroactive ligand receptor interaction	2	0.5909	1.1584	0.0309	C3AR1, FPR2
*LINC00937*					
Apoptosis	3	−0.7738	−1.6314	0.0036	IRAK3, CFLAR, NFKBIA
Toll like receptor signaling pathway	6	−0.4242	−1.2428	0.0178	NFKBIA,MAPK14,TLR8,TLR5,TLR4,TLR2
Neurotrophin signaling pathway	3	−0.5119	−1.0841	0.0353	IRAK3,NFKBIA,MAPK14
Cytokine-cytokine receptor interaction	4	0.6016	1.4480	0.0080	IL1R2,IL18RAP,IL18R1
MAPK signaling pathway	3	0.5000	0.9982	0.0489	IL1R2,MKNK1,MAPK14

### Expression level analysis for hub gens

**Fig. 7:**
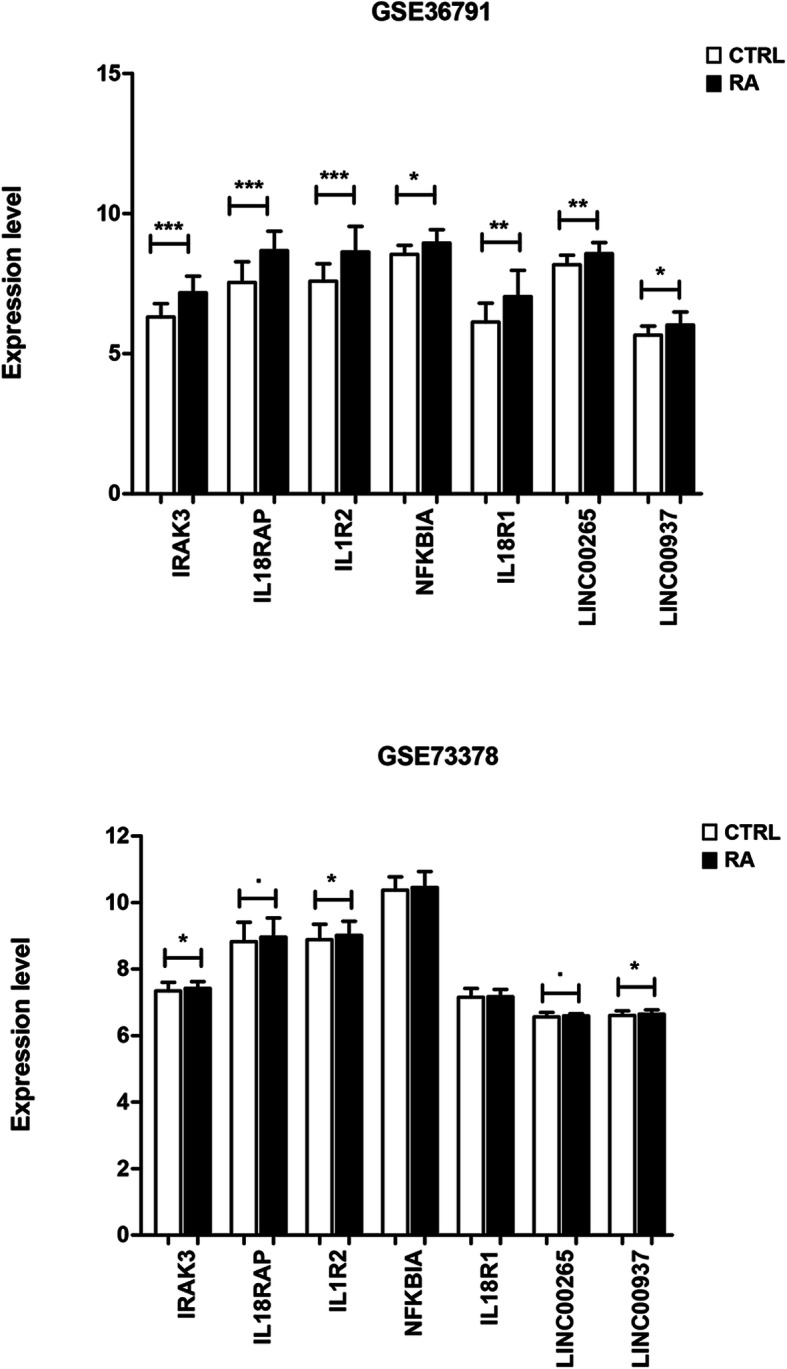
Comparison of expression levels of hub genes in different sample groups. Up: GSE36791. Down: GSE73378. White square: The average expression level of genes in the CTRL sample group. Black square: The average expression level of genes in the SHA patients form RIA sample group

We compared the expression level of the seven genes in different samples. As is shown in Fig. 7, the expression trend is consistent in the two data sets.

## Discussion

SAH caused by RIA is one of the common critical illnesses in cerebrovascular disease. The disability rate among surviving patients is approximately 30% [24]. Bioinformatics was used to analyze the datasets of the RIA and identify potential biomarkers, which is very helpful for clinical diagnosis. Wang et al. identified six hub genes associated with rupture of intracranial aneurysms by weighted gene co-expression network analysis [25]. Wan et al. identified 4 hub genes. Represent potential biomarkers of SAH or impending the likelihood of IA progression and rupture [26]. hsa-miR-1304, hsa-miR-33b, hsa-miR-125b, and hsa-miR-125a-5p were predicted to take part in the pathogenesis of SAH caused by RIA [27]. At present, lncRNA has gradually received attention. Its functions are complex and can participate in the various stages of regulating gene expression.

In previous research, Pera et al. proved that RIA strongly influences the transcriptional profiles of peripheral blood cells [13]. They found 16 genes and lymphocyte-to-monocyte-and-neutrophil gene expression ratios distinguished RIA patients from normal. In our research, we want to explore some hub lncRNAs related to SHA. Five hundred sixty-one DERs, including 25 DElncRNAs and 536 DEmRNAs were identified between SAH group and normal controls and the functional analyses of DEmRNAs showed that they were dramatically enriched in GO-BP terms of immune response and KEGG pathway of T cell receptor signaling pathway. Moreover, the WGCNA module analysis revealed that six modules significantly positively correlated with SAH, which includes 217 DERs (2 lncRNAs and 215 mRNAs). Additionally, lncRNA and mRNA regulatory network contained 240 lncRNA-mRNA interaction pairs among 169 DEmRNAs and 2 DElncRNAs (LINC00265 and LINC00937). The GSEA analysis revealed that LINC00265 and LINC00937 all played essential roles in cytokine-cytokine receptor interaction, neurotrophin signaling pathway, and apoptosis pathways, and these three pathways were found to be implicated with SAH development and suggested five genes (NFKBIA, IRAK3, IL1R2, IL18RAP, and IL18R1) exhibited strong correlation with them. We compared the expression levels of these genes between different samples in data sets GSE36791 and GSE7337. The two data sets show the same expression trend.

A previous study revealed that RIA was preferentially associated with increased cell apoptosis levels and proposed that apoptosis might decrease the resistant ability of aneurysm wall, leading to its rupture [28]. Overwhelming evidence has demonstrated that early brain injury (EBI) largely contributes to elevated mortality risks within 24–72 h after RIA and neuronal apoptosis is responsible for EBI [29, 30]. Yuksel et al. pointed out that apoptotic cell was observed in cortical, subcortical or hippocampal neurons after SAH and cell apoptosis exerted critical roles in EBI, which suggested that apoptosis may be a research agenda for clarifying the mechanisms of SAH from RIA [31]. Herein, we found that LINC00265/LINC00937-*NFKBIA* was predominantly related to cell apoptosis. Increasing studies have reported that that activation of NFKB pathway is the main cause of EBI occurring following SAH. Conversely, its inhibition had neuroprotective roles against SAH [32, 33]. Interestingly, Zhang et al. argued that inhibiting TGFβ-activated kinase 1 significantly suppressed NFKB activity, reduced neuronal apoptosis in SAH [34]. In neurons or cerebral tissue, NFKB signaling pathways could activate the anti-apoptotic proteins (Bcl-2 and Bcl-xL) [35]. In addition, NFKB also participates in regulating the inflammatory responses. NFKB activation could enhance gene expression level of many inflammatory factors such as IL-1β, which may accelerate the cell death in the brain [36]. You et al. reported that NFKB was activated in the neurons following SAH and regulated inflammatory genes expression, thereby causing delayed brain injury [37]. Our finding showed that a NFKB inhibitor gene, *NFKBIA* was increased in blood samples of SAH patients compared with healthy controls. The anti-inflammatory effect of *NFKBIA* in SAH needs to be investigated in the following analysis. Our results also showed that there were strong associations between LINC00265/LINC00937-*NFKBIA* and neurotrophin signaling pathway. Notably, the dysgregulation of neurotrophin was correlated with neuron survival and death. For example, the binding of neurotrophin to p75 neurotrophin receptor (p75NTR) is necessary for neuron apoptosis [38]. Recently, the interaction between p75 neurotrophin receptor (p75NTR) and the pro-apoptotic BH3-only protein NIX was identified by Shen et al., who argued that the interaction of p75NTR and protein NIX were crucial for the p75NTR-mediated neuron apoptosis in intracerebral hemorrhage [39]. Many studies have also highlighted that neurotrophic factors were crucial for the neurological recovery following SAH [40, 41]. A research showed that oxidized high-mobility group box1 protein dramatically facilitated brain recovery through increasing the expression of neurotrophin in the late stage of SAH, which implied that neurotrophin pathway activation was a key pathological processe in SAH [42]. Therefore, we inferred that LINC00265/LINC00937-*NFKBIA* might be implicated with the underlying pathogenesis of SAH from RIA via apoptosis and neurotrophin signaling pathway. *IRAK3*, another NFKB inhibitor was also upregulated in SAH patients compared to healthy controls. The co-expression analysis indicated that LINC00265 strongly interacts with *IRAK3*. This gene also participated in neurotrophin signaling pathway and apoptosis. Although few studies explored the potential molecular mechanisms with the involvement of *IRAK3* in SAH from IAR and there was no direct evidence involving the underlying roles of LINC00265/LINC00937 in SAH, our results provide a new clue that LINC00265-*IRAK3* might be involved in the pathogenesis of SAH from RIA by inhibiting *NFKB*.

## Conclusion

This study identified 2 lncRNAs and 5 genes from SAH patients with RIA, which may enhance our current knowledge on this disease and may provide potential biomarkers of this disease. They may be involved in the pathogenesis of SAH from RIA by activating neurotrophin signaling pathway and apoptosis.

## Supplementary Information

**Additional file 1: Supplementary Table S1.** The list of significantly differentially expressed RNAs (DERs) (lncRNA and mRNAs) in SAH samples relative to controls.

**Additional file 2: Supplementary Table S2.** The list of sample information.

**Additional file 3: Supplementary Table S3.** The list of significantly differentially expressed RNAs (DERs) included in the 6 WGCNA modules.

**Additional file 4: Supplementary Table S4.** The list of the lncRNA-mRNA pairt with Pearson correlation coefficients (PCC) higher than 0.6.

## Data Availability

The datasets analysed during the current study are available in the National Center for Biotechnology Information-Gene Expression Omnibus (NCBI-GEO) database (https://www.ncbi.nlm.nih.gov/geo/). We downloaded two datasets: GSE36791 and GSE7337.

## Abbreviations

SAH: Subarachnoid hemorrhage
WGCNA: Weighted gene co-expression network analysis
NFKBIA: NF-κB inhibitor alpha gene
eNOS: Endothelial nitric oxide synthase
lncRNA: Long non-coding RNA
GO: Gene Ontology
KEGG: Kyoto Encyclopedia of Genes and Genomes
GSEA: Gene Set Enrichment Analysis
